# New Genera and Species of *Caulobacter* and *Brevundimonas* Bacteriophages Provide Insights into Phage Genome Evolution

**DOI:** 10.3390/v16040641

**Published:** 2024-04-20

**Authors:** Bert Ely, Michael Hils, Aaron Clarke, Maegan Albert, Nadia Holness, Jacob Lenski, Tannaz Mohammadi

**Affiliations:** Department of Biological Sciences, University of South Carolina, Columbia, SC 29208, USAclarke1016@gmail.com (A.C.); maeganc.albert@gmail.com (M.A.); tannaz@email.sc.edu (T.M.)

**Keywords:** bacteriophages, *Caulobacter*, *Brevundimonas*, *Autographiviridae*, genome comparisons, indels, new genera and species

## Abstract

Previous studies have identified diverse bacteriophages that infect *Caulobacter vibrioides* strain CB15 ranging from small RNA phages to four genera of jumbo phages. In this study, we focus on 20 bacteriophages whose genomes range from 40 to 60 kb in length. Genome comparisons indicated that these diverse phages represent six *Caulobacter* phage genera and one additional genus that includes both *Caulobacter* and *Brevundimonas* phages. Within species, comparisons revealed that both single base changes and inserted or deleted genetic material cause the genomes of closely related phages to diverge. Among genera, the basic gene order and the orientation of key genes were retained with most of the observed variation occurring at ends of the genomes. We hypothesize that the nucleotide sequences of the ends of these phage genomes are less important than the need to maintain the size of the genome and the stability of the corresponding mRNAs.

## 1. Introduction

Bacteriophages are viruses that infect bacteria, and they are considered potential biological control agents for bacteria due to their generally narrow host cell specificity. Importantly, bacteriophages, also known as phages, are found in almost all environments including soil, fresh and marine water samples, and most recently in the free atmosphere [1]. They are exceptionally numerous and are approximately ten times more abundant than their bacterial hosts [2]. They are present wherever bacteria exist and play an important role in the ecology and evolution of their bacterial hosts [3]. Nevertheless, much of the vast structural and genomic diversity of bacteriophages is still to be elucidated [4].

Extensive studies of the dimorphic bacterium *Caulobacter vibrioides* have made it the best studied Alphaproteobacterium. It has an unusual cell cycle since each cell division results in a mature stalked cell and an immature swarmer cell. The stalked cell can start a new round of cell division immediately, but the swarmer cell must mature into a stalked cell before it can undergo cell division. The motile swarmer cell has a flagellum and pili that make it susceptible to the flagellotropic Cbk-like phages and RNA phages that attach to the pilus [5,6,7]. Since the stalked cell has neither of these appendages, it is resistant to these bacteriophages. Thus, the phage-resistant stalked cell will generate a continual supply of newly generated host cells for the swarmer cell-infecting phages. Other types of *Caulobacter* phages attach to both cell types [5,8]. *Brevundimonas* is a morphologically similar sister genus to *Caulobacter* [9]. However, most species do not grow stalks, and they differ from *Caulobacter* in lipid composition.

Building on the foundational work of Johnson and Ely [5], our laboratory has initiated a project to isolate and characterize large numbers of new *Caulobacter* phages to document the large variety of naturally occurring bacteriophages that can infect members of the genus *Caulobacter*. This effort has been facilitated by our observation that *C. vibrioides* strain CB15 is sensitive to many more phages than any other *Caulobacter* strain that our laboratory has tested. Using this host strain, we characterized a newly isolated virus designated Lullwater that was distantly related to two previously described *Caulobacter* viruses, Cd1 and Percy [8]. The genomes of all three phages coded for single subunit RNA polymerases suggesting that they might be members of the large *Autographiviridae* family. A striking feature of these genomes was that they shared many of the same genes, and the order and orientation of these genes was conserved. However, among the shared genes, the predicted amino acid identities were often less than 80% suggesting a distant relationship. Viral genera are now defined by the International Committee for the Taxonomy of Viruses (ICTV) as taxonomic groupings of viruses that have genomes with nucleotide identities greater than or equal to 70% [10]. The genome nucleotide identities are calculated by multiplying the percent identity observed in pairwise genome nucleotide comparisons by the extent of the query coverage. Based on these criteria, the Lullwater, Percy, and Cd1 phages should be classified as members of three distinct genera.

Subsequently, we described three additional *Caulobacter* phages that are distantly related to the *Rauchvirus* BPP-1 [11]. However, based on the current ICTV guidelines, these three *Caulobacter* phages are too distantly related to BPP-1 to be considered part of the *Rauchvirus* genus. Two of these phages, JessA and SR18, are closely related members of the newly proposed *Jessavirus* genus, and the third, RW, is a member of a separate genus that we have tentatively designated *Riverwalkvirus*.

In this paper, we describe six newly isolated *Caulobacter* phages and two *Brevundimonas* phages that are members of the large *Autographiviridae* family and compare them to the Lullwater, Percy, and Cd1 phages described above [8] and a recently described *Brevundimonas* phage [12]. In addition, we describe eight additional phages that are members of the proposed *Jessavirus* and *Riverwalkvirus* genera. Using the recently published criteria for defining species and genera [10], we classify these 20 bacteriophages into seven genera. Furthermore, within species comparisons reveal that these phage genomes diversify by the loss or addition of new genetic material as well as the accumulation of mutations that cause single base changes.

## 2. Materials and Methods

### 2.1. Isolation of Bacteriophages

The phages described in this study were isolated from water samples using the enrichment procedure described by Nguyen and Ely [8]. Briefly, 10 mL of a water sample from a stream or pond was filtered, mixed with 2.5 mL 5X PYE culture medium, 0.625 mg streptomycin sulfate, and 100 µL of a culture of SC1004, a streptomycin-resistant mutant of the *C. vibrioides* CB15 strain [13]. After overnight incubation at 29 ^o^C, the lysate was centrifuged to remove most of the bacteria and then a small amount of chloroform was added to lyse any remaining bacteria. Finally, 10 µL of the enriched sample was spotted onto a PYE plate overlaid with 3.5 mL of PYE agar containing 100 µL of a fresh culture of the SC1004 host strain. If lysis was observed after overnight incubation, the sample was diluted and replated to obtain single plaques. Subsequently, representative plaques were chosen for further purification so that a lysate derived from a single phage particle could be obtained. Most of the water samples tested were from a single sampling site on a portion of the Rocky Branch Creek that crosses the University of South Carolina campus.

An alternate bacteriophage host, BRV2, was a bacterial strain isolated from the roots of a plant growing on the University of South Carolina Columbia campus. After isolating DNA from BRV2, PCR using 16S rRNA primers followed by Sanger sequencing revealed that the strain belonged to an unknown species of the *Brevundimonas* genus. A streptomycin-resistant derivative of BRV2 designated SC4334 served as the host for two independent phage isolates designated AA and BC isolated from Congaree River (Columbia, SC, USA) water samples.

### 2.2. Host Range

Host range was determined by mixing 100 µL of a potential host culture with 3 mL of melted PYE soft agar [5] and pouring the mixture onto the surface of a PYE plate. After the top layer cooled and solidified, 10 µL aliquots of various dilutions of the phage lysates were spotted on the surface. After overnight incubation at 32 °C, each spot was examined for the ability to produce a clear zone indicating that the phage could lyse the potential host cells. The host strains tested were *C. vibrioides* strains CB15, CB2, CB13; *C. segnis* strains CBR1 and TK0059; and finally, the FWC26 and ME4 *Caulobacter* strains that belong to a third species that is closely related to both the *vibrioides* and *segnis* species.

### 2.3. Genome Nucleotide Sequence Determination

Genomic DNA was isolated from phage lysates using a Qiagen DNA Isolation Kit according to the manufacturer’s instructions. DNA sequencing was performed by the University of Florida or the University of Maryland sequencing facilities and assembled using the HGAP4 assembly program. The assembled genomes were aligned with the genomes of similar phages using Mauve [14], and excess sequence information was removed to generate unit length genomes. The final genome sequence was annotated with RAST (http://rast.nmpdr.org) and then edited manually in Artemis [15] to match the annotation of closely related phage genomes. Genome nucleotide sequence comparisons were performed using Viridic [16]. All genome sequences were deposited in GenBank (https://www.ncbi.nlm.nih.gov/).

The GenBank accession numbers are: Lullwater, MF621978; BL94, OQ135105; BL198, OQ135102; Percy, KT381879; BL199, OQ135103; DCM, OQ137559; KSC, OQ135104; ERS, OQ137560; AA, OK319016; BC, OK335783; Cd1, GU393987; JessA, MK942064; SR18, MN746332; RapA, OQ137562; Quill, OR260090; RW, MK929790; RLK, OQ135106; GB2A, OR260089; KcrB, OQ137561; Babayka, ON529868. 

## 3. Results and Discussion

**Figure 1:**
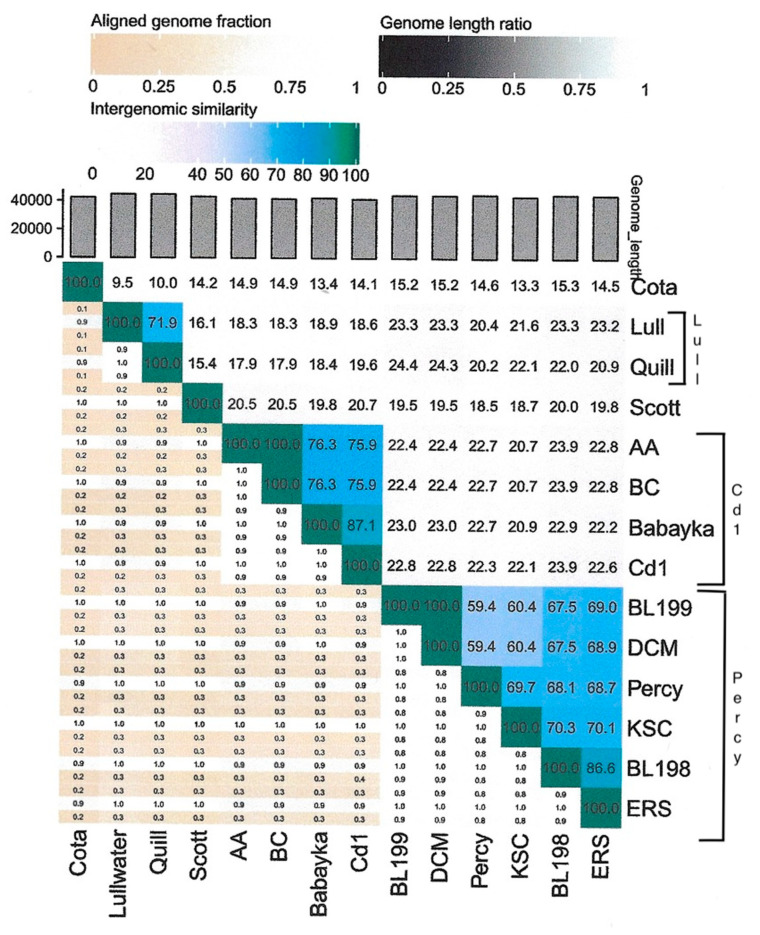
Viridic [16] heatmap of *Caulobacter* and *Brevundimonas* bacteriophages in the *Autographiviridae* family. The Lullwater (Lull), Cd1, and Percy phage genome clusters are indicated with brackets. *Sphingomonas* phage Scott and *Xylella* phage Coda were included as outgroups.

**Figure 2:**
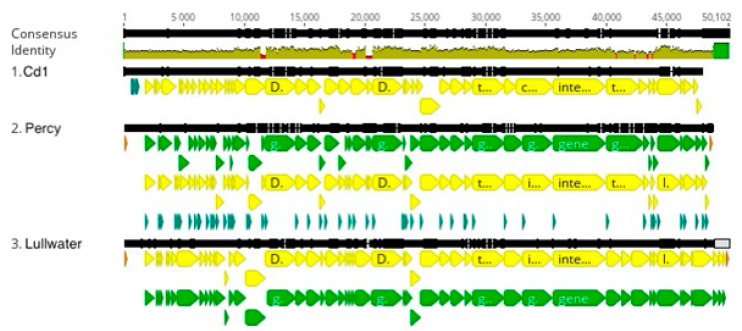
An alignment of a representative genome from each of the three *Autographviridae* clusters. The black lines indicate the position in the genome and the brown line is a sliding scale of the genome nucleotide consensus identity. The green lines indicate the position of genes, and the yellow lines indicate the positions of CDS. The red sections in the olive-colored consensus identity bar represent the positions of insertions or deletions (indels).

As part of our laboratory’s long-term sampling project, 11 new *Caulobacter* phages (BL94, BL198, BL199, DCM, ERS, KSC, GB2A, RLK, KcrB, RapA, and Quill) and 2 *Brevundimonas* phages (AA and BC) have been isolated and characterized. After determining the nucleotide sequence of these 13 phage genomes, we compared the amino acid sequences of the major capsid genes to the GenBank database to identify the closest relatives. Eight of these new phage genomes coded for a single subunit RNA polymerase indicating that they belonged to the *Autographiviridae* family, and the five remaining phages were related to either the previously described JessA or RW [8] phages. Based on this analysis, we used Viridic [16] to determine the degree of genetic relatedness among 14 members of the *Autographiviridae* family (Figure 1). Two of these phages, Cota and Scott, were used as outgroups and infected *Xylella* and *Sphingomonas* strains [17], respectively. Based on these results, we grouped the 12 *Caulobacter* and *Brevundimonas* phage genomes into three clusters of related phages designated the Lullwater, Cd1, and Percy clusters. An alignment of a representative of each of these clusters showed that the gene arrangements were conserved with the positions of all the larger genes and many of the smaller genes at the same location in all three genomes (Figure 2). The red sections in the olive-colored consensus identity bar represent the positions of insertions or deletions (indels). 

### 3.1. The Lullwater Cluster

The 45 kb Lullwater phage genome codes for 52 proteins and has a 196 bp terminal repeat [8]. Recently, we determined the genome sequence of a related phage, designated Quill, that codes for 53 proteins. In both phage genomes, the same 23 proteins have annotated functions (Table 1). However, since the intergenomic similarity of these two phage genomes is only 71.9% (Figure 1), they should be considered members of two different species of the proposed Lullwatervirus genus according to the guidelines established by the International Committee for the Taxonomy of Viruses (ICTV) [9]. Despite these genomic sequence differences, both phages can infect the same sets of *C. vibrioides* and *C. segnis* wild type test strains (Table 2).

**Figure 3:**
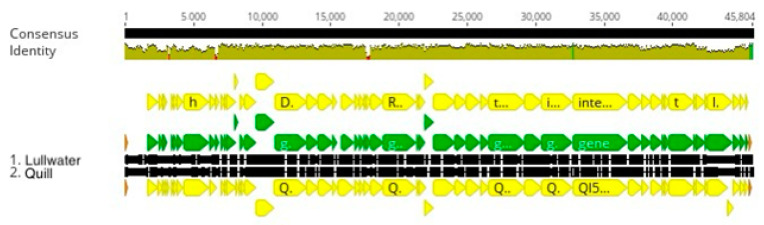
Lullwater cluster genome alignment. The black lines indicate the position in the genome and the brown line is a sliding scale of the genome nucleotide consensus identity. The green lines indicate the position of genes, and the yellow lines indicate the positions of CDS. The red sections in the olive-colored consensus identity bar represent the positions of insertions or deletions (indels).

A comparison of the Quill and Lullwater genomes shows that the ends of the genomes are more variable than the central 32 kb (Figure 3). In addition, there are multiple indels in the first 6 kb and the last 6 kb but only one indel in the central portion of the genomes. Thus, the ends of these two genomes seem to be more prone to single base changes and indels than the large interior region. Since the interior of these phage genomes contain all the structural and replication genes, there would be selective pressure to conserve these regions of the genome. In contrast, the beginning of the two genomes contains only hypothetical genes, and the end contains hypothetical genes plus the conserved terminase and lysis genes. Thus, selective pressure in these regions is likely to be much weaker than that in the central region.

### 3.2. Cd1 Cluster

*Caulobacter* phage Cd1 was one of the first *Caulobacter* phage genomes to be sequenced [19], and to date, we have not found any additional *Caulobacter* phages that are closely related to Cd1. However, we recently isolated two phages that infect *Brevundimonas* strain BRV2 and are related to Cd1 based on their genome sequences (Figure 1). *Brevundimonas* is a sister genus to *Caulobacter* [9], and BRV2 is a rod-shaped bacterium that produces flagellated swarmer cell progeny that are morphologically similar to those produced by *Caulobacter*. Bacteriophage AA was isolated from a water sample taken from the Congaree River, and BC was isolated from a water sample taken from a nearby stagnant puddle using the streptomycin-resistant BRV2-derivative SC4334 as the host strain for both phages. Host range experiments showed that both AA and BC produced plaques on soft agar lawns containing *Brevundimonas* strains BRV2 and DS20, but they did not lyse the other *Brevundimonas* strains tested (BRV3 and ME6) or any representatives of six different *Caulobacter* species (Table 2). In contrast, Cd1 was able to infect both the *C. vibrioides* and the *C. segnis* wild type strains but not the *Brevundimonas* strains.

**Figure 4:**
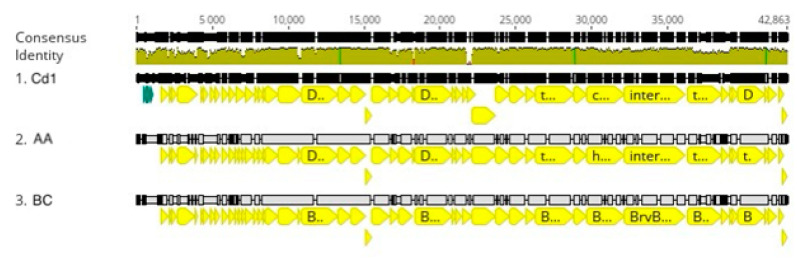
Cd1 cluster genome alignment. The black lines indicate the position in the genome and the brown line is a sliding scale of the genome nucleotide consensus identity. The yellow lines indicate the positions of CDS. The red sections in the olive-colored consensus identity bar represent the positions of insertions or deletions (indels).

The AA and BC genomes contain 42 kbp of DNA, and each genome codes for 50 proteins including 22 with annotated functions. These 22 annotated genes correspond to all the genes that are annotated in the Lullwater genome except the nucleotidyltransferase gene (Table 1). Each of the genes is transcribed from the same strand of DNA, and the close stop and start codon spacing suggests that all but seven genes might be transcribed as one or two long transcripts (Figure 4). When the nucleotide sequences of the AA and BC genomes were compared, the two sequences differed by a single-base pair in the tail fiber gene that changes a proline codon in the AA gene to a glutamate codon in the BC gene.

Recently, a third related *Brevundimonas* phage designated vB_BpoS-Babayka was isolated with *Brevundimonas pondensis* LVF1 as its host [12]. Based on the published annotation, this phage codes for 53 proteins. However, the vB_BpoS-Babayka genome deposited at NCBI contains an annotation error that does not include the beginning of the DNA polymerase gene. The corrected annotation shows that the DNA polymerase gene begins at base 2036 and is transcribed in the reverse direction past the beginning of the genome and ending with the coding region correctly annotated at the other end of the genome. This corrected annotation also eliminates three hypothetical genes at the beginning of the genome bringing the total number of annotated genes down to 50.

When the AA phage genome was compared to the Babayka genome, the two genomes had an intergenomic similarity of 76.3% (Figure 1). Thus, the two phages should be considered members of different species within the proposed Congareevirus bacteriophage genus. Interestingly, the closest match to the Babayka *Brevundimonas* phage genome was the *Caulobacter* bacteriophage Cd1 genome with an intergenomic similarity of 87.1%. Thus, Babayka is more closely related to the *Caulobacter* phage Cd1 than it is to the other *Brevundimonas* phages. Despite these differences, nearly all the genes are at the same relative genome locations in all four phage genomes. Finally, based on current recommendations for virus taxonomy [10], the AA and BC phages and the Babayka phage represent two new species of bacteriophages in the same genus as phage Cd1 [20]. Despite these similarities, the Cd1 genome contains four genes that code for hypothetical proteins that are not present in the AA and BC genomes, and the AA and BC genomes contain three genes that code for hypothetical proteins that are not present in the Cd1 genome (Figure 4). Likewise, the Cd1 genome contains one gene that codes for a hypothetical protein that is not present in the Babayka genome, and the Babayka genome contains one gene that codes for a hypothetical protein that is not present in the Cd1 genome. As with the other phage clusters, most of these gene differences occur at the ends of the phage genomes. In addition, as expected, the genes that code for the AA and BC tail fiber proteins are quite different from the corresponding genes in the Babayka and Cd1 genomes (Figure 4, position 35,300 to 37,300) reflecting the differences in host specificity.

### 3.3. Percyvirus Cluster

Recently, we isolated and characterized five additional *Caulobacter* phages—BL198, BL199, DCM, KSC, and ERS—that are more closely related to Percy than to the more distantly related Lullwater and Cd1 phages (Figure 1). In addition, genome alignments indicated that approximately 90% of the genes in the Percy genome were present in each of the other five phage genomes. Genome sizes of these five phages range from 43,647 bp to 44,974 bp, and they code for 53 to 57 genes. For most of the phages, the host range includes both the *C*. *vibrioides* and the *C. segnis* test strains. However, the ERS and KSC phages have a more limited host range since ERS does not infect the *C. segnis* strain CBR1, and KSC does not infect either of the *C. segnis* test strains (Table 2).

According to the guidelines established by the ICTV [10], phage genomes in the same species would need to have an intergenomic similarity of 95%. Based on this criterion, only BL199 and DCM would be considered members of the same species, and indeed, they differed by only eight single base mutations and an indel. Similarly, according to the ICTV guidelines, two viral genomes should have at least 70% intergenomic similarity to be members of the same genus. Based on this criterion, the BL199 and DCM species would be in the proposed Dcmvirus genus that is distinct from the other genera defined for this cluster. Similarly, KSC, BL198, and ERS would be considered members of three separate species within a second phage genus designated Columbiavirus, and Percy would be the sole current representative of the Percyvirus genus. Thus, although the Lullwater and Cd1 phage clusters represent multiple species within a single genus, the Percy cluster is more diverse and consists of three related genera.

**Figure 5:**
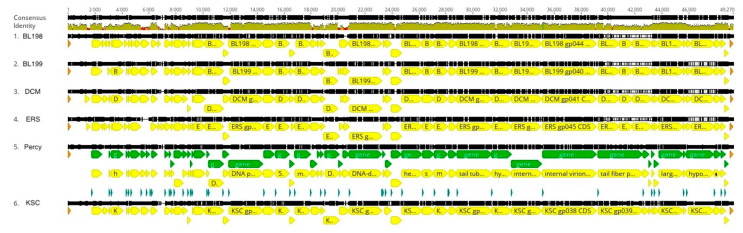
Percy cluster genome alignment. The black lines indicate the position in the genome and the brown line is a sliding scale of the genome nucleotide consensus identity. The green lines indicate the position of genes, and the yellow lines indicate the positions of CDS. The red sections in the olive-colored consensus identity bar represent the positions of insertions or deletions (indels).

Despite this level of genomic nucleotide sequence diversity, the order and presence of genes is highly conserved with all genes being transcribed in the same direction (Figure 5). Exceptions to this conservation occur at the ends of the genomes where numerous differences in gene annotation occur and indels cause some genes to be present in some genomes but missing in others.

### 3.4. Jessavirus Cluster

**Figure 6:**
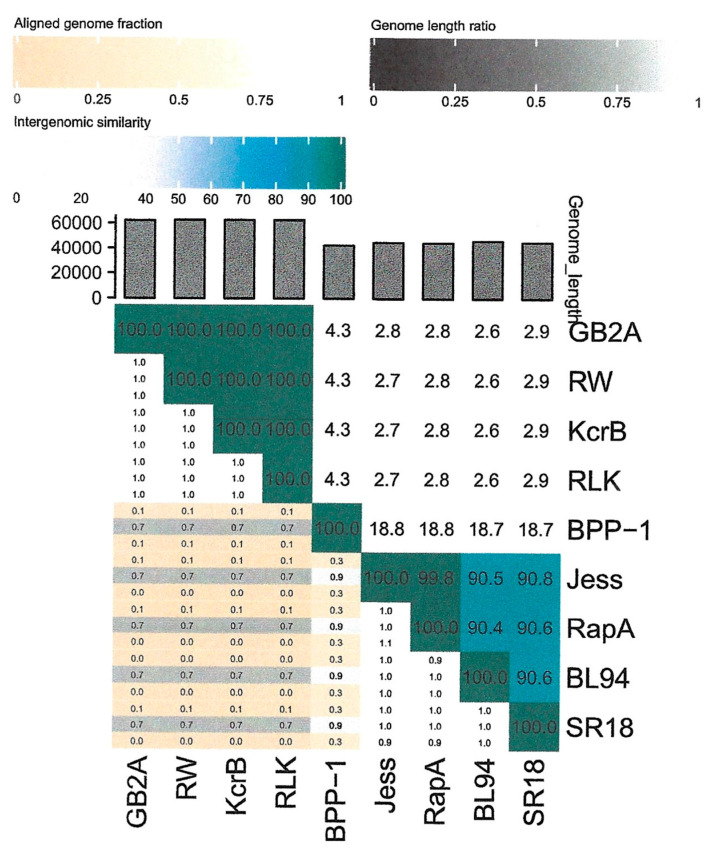
Viridic [16] heatmap of *Caulobacter* bacteriophages belonging to the Jessavirus (defined by the lower black and blue boxes) and Riverwalk clusters (defined by the upper black boxes). *Bordetella* phage BPP-1 was included as an outgroup.

**Figure 7:**
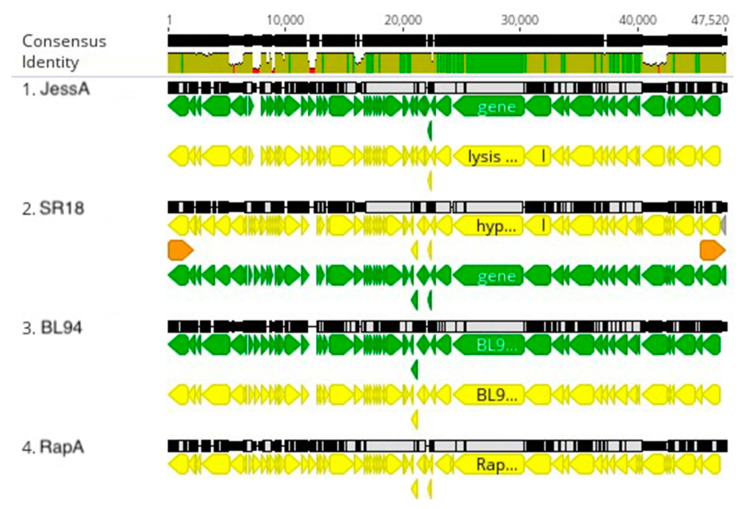
JessA cluster genome alignment. The black lines indicate the position in the genome and the brown line is a sliding scale of the genome nucleotide consensus identity. The green lines indicate the position of genes, and the yellow lines indicate the positions of CDS. The red sections in the olive-colored consensus identity bar represent the positions of insertions or deletions (indels).

The JessA, SR18, RapA, and BL94 *Caulobacter* phages are only distantly related to the Cd1, Lullwater, and Percy clusters, and they do not code for an RNA polymerase. Therefore, they are not members of the *Autographiviridae* family. Host range experiments showed that all the JessA cluster phages can infect both the *C. vibrioides* and the *C. segnis* wild type strains (Table 2). When the nucleotide sequences of the four genomes were compared, the JessA and Rap A genomes were nearly identical (Figure 6 and Figure 7). In contrast, pairwise whole genome nucleotide sequence comparisons indicate that SR18 has 90.8% and 90.6% intergenomic similarity with JessA and RapA, respectively (Figure 6). Thus, SR18 is sufficiently different from JessA and RapA to be considered a new species relative to the other two. However, both species should be considered members of the proposed Jessavirus genus. When the SR18 genome was compared to the JessA genome, multiple indels were observed at the ends of the genomes, but not in the large central portion of the two genomes (Figure 7).

BL94 contains three genes coding for hypothetical proteins that are not present in the JessA genome, and the JessA genome contains five genes coding for hypothetical proteins that are not present in the BL94 genome, with all these gene differences occurring near the ends of the phage genomes (Figure 7). In addition, the genomic GC content of BL94 is 64.5% compared to 64.2 and 64.3% for JessA and SR18, respectively. Although these differences in the number of genes and genomic GC content are small, they illustrate the extent of the evolutionary divergence of these three bacteriophage genomes. These differences are sufficiently large that all three phages should be considered members of different species in the proposed Jessavirus genus (Figure 6). However, one shared gene of particular interest in the BL94 genome codes for a large lysis protein (designated lysis in Figure 7) that has only three SNPs near the beginning and one SNP near the end of the 6120 bp gene compared to the corresponding gene in JessA. Thus, this gene is more highly conserved between these two genomes than would be expected from the overall 90.8% average nucleotide identity.

### 3.5. Riverwalk Cluster

The size of the previously described RW genome [11] is 62 kbp, and it contains a tRNA^val^ gene and 105 protein coding genes, only 22 of which have functional annotations. We previously showed that the relative positions and orientation of the DNA replication and packaging genes were conserved between the RW and JessA genomes [11]. However, a more careful analysis revealed that only the DNA polymerase and helicase genes code for proteins with more than 45% amino acid sequence similarity (47% and 53%, respectively). In addition, the RW DNA polymerase gene contains a 720 bp sequence that is not present in the JessA DNA polymerase gene so the RW DNA polymerase protein would contain 240 additional amino acids near the C terminus of the protein. The lack of additional homologous DNA sequences and the 17 Kb difference in genome size would indicate that the Riverwalk cluster phages are not closely related to the JessA cluster phages, and the predicted amino acid sequence similarity of the helicase and DNA polymerase genes may be a product of functional selection.

**Figure 8:**
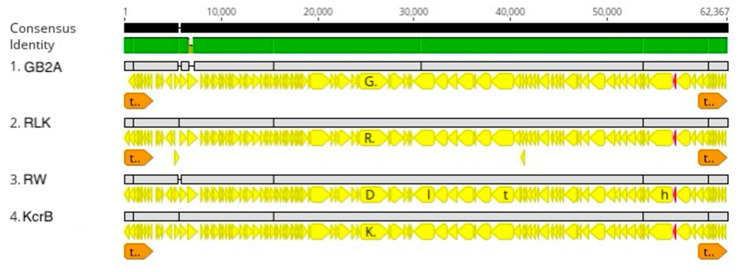
RW cluster genome alignment. The black lines indicate the position in the genome and the green line is a sliding scale of the genome nucleotide consensus identity. The yellow lines indicate the positions of CDS.

In addition to RW, we have isolated three similar phages, RLK, KcrB, and GB2A. All four phages can infect the *C. vibrioides* wild type strains, but not the other *Caulobacter* wild type strains that we tested (Table 2). The RLK and KcrB genomes differ from that of RW by four single-base pair changes and three single-base pair changes, respectively, while KcrB differs from RLK at one single-base pair (Figure 8). The GB2A genome differs from RW by two single-base pair changes, two additional Cs in an intergenic region, and a 375 bp deletion in the gene for a pentapeptide repeat protein. Therefore, all four phages are closely related isolates of the same species of phage in the proposed Riverwalkvirus genus (Figure 5). However, the RLK and KcrB phages were isolated from two different places in Minnesota, and the RW and GB2A phages were isolated from two separate locations in Columbia, SC. These results suggest that the genomes of the Riverwalkvirus genus are stable since very few differences are observed in the genomes of these pairs of phages that were isolated from locations that were more than 1000 miles apart. In fact, we found more genetic variation among a set of seven closely related members of a new species of the *Dolichocephalovirinae* subfamily isolated independently from a single Columbia, SC sampling site than we found among the Riverwalk cluster of phages.

The genome organization of the RW cluster is interesting. Based on the lack of spaces between genes, a long operon that starts on the 5′-side of the tRNA gene and close to genome position 56,736 and ends just after RW position 29,945 codes for a long string of hypothetical proteins followed by the genes involved in building the tail structure. A second long operon appears to start near genome position 7862 and ends just after position 29,830. It also starts with a long string of genes that code for hypothetical proteins, and then additional genes that code for the proteins that are involved in DNA replication. The gene that codes for the major capsid protein has not been identified in the genomes of the RW cluster.

## 4. Conclusions

In this study, we describe the isolation and genome sequencing of 13 additional bacteriophages that are related to seven previously described *Caulobacter* and *Brevundimonas* phages. Using the new ICTV criteria [10], we grouped these phages into six *Caulobacter* phage genera and one new genus that includes both *Caulobacter* and *Brevundimonas* phages. Furthermore, genome comparisons of closely related phages illustrated that single base changes and indels are the primary contributors to the genetic diversity that we observed within and between phage species. We also showed that some phage species genomes tend to be highly conserved, while others diversify more rapidly.

Interestingly, the genome diversification that we observed with these phages occurred more rapidly in the terminal regions than it did in the central regions where the structural and DNA replication genes are located. This result suggests that the selective pressure to provide functional proteins for genome replication and the assembly of functional phage capsids not only selects against single-base changes, but also the introduction of indels that might disrupt gene organization and expression. Conversely, the data also suggest that the genes and genome arrangements at the ends of these phage genomes are much less constrained. In fact, it is possible that many of the proteins transcribed from these terminal regions do not serve a useful purpose. For example, the primary purpose of small peptides may be that their translation keeps the ribosomes on the mRNA to protect the mRNA and make sure that the distally located larger proteins are translated with fidelity at the rate needed for the generation of new phage particles. In addition, the size of the terminal regions may be important for maintaining a properly sized genome that can be packaged into the phage head capsule. The fact that the new segments of DNA that we observed in these regions seem to be from unknown sources would support the idea that the nucleotide sequences of the genes in the terminal regions may be less important.

Taken together, our comparisons of closely related phage genome sequences provide glimpses into the ways that bacteriophage genome evolution has occurred in natural environments. Importantly, we found similar patterns of phage genome evolution among a wide range of phages with T7-like genomes.

## Figures and Tables

**Table 1:** Phage genome protein annotations. A + indicates that the corresponding gene is present in the phage genome.

	Lullwater, Quill	Cd1, AA, BC, Babayka	Percy, BL198, BL199, DCM, KSC	JessA, RapA, BL94, SR18	RW, RLK, KcrB, GB2A
tRNA nucleotidyltransferase	+	+	+	(BL94 only)	
Protein kinase		+			
DNA primase	+	+	+	+	+
DNA helicase	+	+	+	+	+
DNA polymerase I	+	+	+	+	+
Pentapeptide repeat protein				+	+
5′-3′ exonuclease	+	+	+		+
DNA endonuclease VII	+	+	+		+
Metallodependent Phosphatase	+	+	+		
DNA ligase	+	+	+		
RNA polymerase	+	+	+		
Protease (2)				+	
Head-to-tail joining protein	+	+	+	+	
Scaffold protein	+	+	+		
Major capsid protein	+	+	+	+	
Tail tubular protein A	+	+	+		
Tail tubular protein B	+	+	+		
Tail tape measure protein					+
Internal virion proteins (2)	+	+	+	+	
Tail fiber protein	+	+	+	+	+
Pin holin	+	+	+		
Homing endonuclease					+
DNA methyl transferase					+
Small terminase subunit	+	+	+	+	+
Large terminase subunit	+	+	+	_+_	+
SAR endolysin	+	+	+		+
I-spanin	+	+	+		
O-spanin	+	+	+		

**Table 2:** Host range data. A + indicates that the host is susceptible to the phage in that row. A - indicates that the host is resistant to the phage in that row. CB15 does not have a type 2 restriction/modification system while the other *Caulobacter* strains that we tested have a functional type 2 restriction/modification system ([18]). Therefore, the plating efficiency is 10 to 100-fold higher with the CB15 host for phages previously grown on CB15. However, when the phages are grown on an alternate host, the plating efficiencies are comparable on all the susceptible hosts.

Host -> Phage	CB15	CB2	CB13	CBR1	TK0059	FWC26	ME4	BRV2 and DS20	BRV3 and ME6
Lullwater	+	+	+	+	+	-	-	-	-
Quill	+	+	+	+	+	-	-	-	-
AA	-	-	-	-	-	-	-	+	-
BC	-	-	-	-	-	-	-	+	-
Cd1	+	+	+	+	+	-	-	-	-
BL199	+	+	+	+	+	-	-	-	-
DCM	+	+	+	+	+	-	-	-	-
Percy	+	+	+	+	+	-	-	-	-
KSC	+	+	+	-	-	-	-	-	-
BL198	+	+	+	+	+	-	-	-	-
ERS	+	+	+	-	+	-	-	-	-
GB2A	+	+	+	+	+	-	-	-	-
RW	+	+	+	+	+	-	-	-	-
KcrB	+	+	+	+	+	-	-	-	-
RLK	+	+	+	+	+	-	-	-	-
JessA	+	+	+	+	+	-	-	-	-
RapA	+	+	+	+	+	-	-	-	-
BL94	+	+	+	+	+	-	-	-	-
SR18	+	+	+	+	+	-	-	-	-

## Data Availability

All genomic sequence data have been deposited in the GenBank database.
